# SGLT2 inhibitors for patients with type 2 diabetes and CKD: a narrative review

**DOI:** 10.1530/EC-23-0005

**Published:** 2023-07-31

**Authors:** Merlin C Thomas, Brendon L Neuen, Stephen M Twigg, Mark E Cooper, Sunil V Badve

**Affiliations:** 1 Department of Diabetes, Central Clinical School, Monash University, Melbourne, VIC, Australia; 2 The George Institute for Global Health, Sydney, NSW, Australia; 3 The University of Sydney School of Medicine, Sydney, NSW, Australia; 4 Department of Endocrinology, Royal Prince Alfred Hospital, Sydney, NSW, Australia; 5 Department of Renal Medicine, St George Hospital, Sydney, NSW, Australia; 6 Faculty of Medicine and Health, University of New South Wales, Sydney, NSW, Australia

**Keywords:** chronic kidney disease, CKD, SGLT2i, sodium–glucose transporter 2 inhibitors, type 2 diabetes, eGFR

## Abstract

Sodium‐glucose co-transporter 2 (SGLT2) inhibitors have recently emerged as an effective means to protect kidney function in people with type 2 diabetes and chronic kidney disease (CKD). In this review, we explore the role of SGLT2 inhibition in these individuals. SGLT2 inhibitors specifically act to inhibit sodium and glucose reabsorption in the early proximal tubule of the renal nephron. Although originally developed as glucose-lowering agents through their ability to induce glycosuria, it became apparent in cardiovascular outcome trials that the trajectory of kidney function decline was significantly slowed and the incidence of serious falls in kidney function was reduced in participants receiving an SGLT2 inhibitor. These observations have recently led to specific outcome trials in participants with CKD, including DAPA-CKD, CREDENCE and EMPA-KIDNEY, and real-world studies, like CVD-REAL-3, that have confirmed the observation of kidney benefits in this setting. In response, recent KDIGO Guidelines have recommended the use of SGLT2 inhibitors as first-line therapy in patients with CKD, alongside statins, renin–angiotensin–aldosterone system inhibitors and multifactorial risk factor management as indicated. However, SGLT2 inhibitors remain significantly underutilized in the setting of CKD. Indeed, an inertia paradox exists, with patients with more severe disease less likely to receive an SGLT2 inhibitor. Concerns regarding safety appear unfounded, as acute kidney injury, hyperkalaemia, major acute cardiovascular events and cardiac death in patients with CKD appear to be lower following SGLT2 inhibition. The first-in-class indication of dapagliflozin for CKD may begin a new approach to managing kidney disease in type 2 diabetes.

## Introduction

At least half of all adults with type 2 diabetes (T2D) have comorbid chronic kidney disease (CKD), with either an elevated urinary albumin excretion rate (uAER) and/or a reduced estimated glomerular filtration rate (eGFR) (1, 2). T2D is the single most common cause of CKD and kidney failure in most developed countries and in many developing countries (3, 4, 5) and continues to increase due to increasing diabetes, population ageing and improved survival (5). The enormous health, societal and economic burden associated with CKD demands that some priority should be given to the prevention and treatment of CKD in people with T2D.

Sodium‐glucose co-transporter 2 (SGLT2) inhibitors have recently emerged as an effective means to protect kidney function in patients with T2D. Although SGLT2 inhibitors achieve urinary glucose wasting and were initially developed as glucose-lowering agents for the treatment of T2D, this represents only one of their potential indications. During recent large cardiovascular outcome trials (CVOTs), it was observed that SGLT2 inhibitors may also have additional benefits for preventing the development and progression of CKD in people with T2D beyond what could be expected from glucose lowering alone (6, 7, 8). Similarly, recent studies undertaken in people with T2D and heart failure (HF) have also observed a slower decline in kidney function in participants receiving SGLT2 inhibitors as well as a lower incidence of CKD and kidney failure (9, 10). Given these observations, as well as the known direct actions of SGLT2 inhibitors in the kidney, several large trials in patients with established CKD have been initiated. Notably, three of these (CREDENCE, DAPA-CKD and EMPA-KIDNEY) were stopped early because of unequivocal evidence of benefit in this setting (11, 12, 13). The SCORED study also showed a positive primary outcome, although it was stopped prematurely for administrative reasons (14). Taken together, these findings strongly support the recent recommendations that SGLT2 inhibitors should be considered as first-line therapy, alongside metformin, for the management of all people with T2D with or at high risk of developing CKD (15). Moreover, because of how common CKD is, and will be in the future, there is also now a strong argument that foundational therapy with SGLT2 inhibitors should be considered for all people with T2D if only to reduce the future incidence and burden of CKD.

In this narrative review, we explore the potential role of SGLT2 inhibition in individuals with T2D and CKD or who are at risk of developing CKD. We examine recent clinical trial data and observational evidence following treatment with SGLT2 inhibitors in patients with T2D and CKD, with a focus on their effects on kidney function, cardiovascular (CV) and HF outcomes and safety in this often-challenging setting. As a narrative review, this publication is based on non-systematic searches of PubMed and the authors’ own expertise and knowledge of the relevant literature, without following a specific protocol or set of predetermined inclusion and exclusion criteria.

## CKD in patients with T2D: state of play

### Risk factors for CKD in T2D

By the time that patients with T2D develop impaired kidney function, there has already been a significant and irreversible loss of nephron mass, which makes a return to normal kidney function impossible, while placing extra functional demands on the remnant kidney that further accelerate kidney function decline, even if the risk factors that established the initial nephron damage are subsequently well controlled. Identifying those individuals at risk of losing kidney structure and function before it is irreversibly gone can be practically achieved by the following:

- Carrying out serial monitoring of the eGFR: those individuals (so-called ‘fast progressors’) who are losing more than 3 mL/min/1.73 m^2^/year (e.g. their eGFR has gone from 80 mL/min/1.73 m^2^ to 70 mL/min/1.73 m^2^ in 3 years) are likely to be at increased risk of developing impaired kidney function.
- Estimating the urinary albumin to creatinine ratio (uACR): those individuals with a uACR > 30 mg/g, and especially >300 mg/g, are at increased risk of developing impaired kidney function.
- Identifying individuals experiencing other complications of T2D (e.g. foot disease, eye disease, CV disease (CVD), erectile dysfunction) who are also at increased risk of developing CKD because of the field nature of end-organ damage in T2D.
- Noting individuals with poor control of their T2D, blood pressure, lipid levels and/or weight are also at increased risk of developing CKD because each of these factors contributes to kidney function decline.
- Recognizing that certain ethnic groups (e.g. individuals from Indigenous Australian, Polynesian, Indian and South-east Asian, Middle Eastern, Hispanic and African backgrounds) and disadvantaged populations also have an increased risk of developing T2D and CKD.
- Understanding that individuals with a strong family history of CKD and kidney failure are at increased risk of developing CKD and impaired kidney function, particularly with the development of T2D.

In each of these settings, the early initiation of multifactorial interventions to slow the decline in kidney function and reduce the development of impaired kidney function (detailed later) should be strongly considered. Other (low-risk) individuals should continue to be monitored on a regular basis for the development of risks or signs of progressive kidney function decline. Some individuals will also have a personal preference for a more proactive approach to their management, including protection of their kidney function even if it currently appears normal (e.g. individuals with a family member with CKD).

### Screening and diagnosis

Because CKD is so common and best detected early, screening for CKD should be undertaken in all people with T2D at the time of their diabetes diagnosis, and at least annually thereafter (15). This testing encompasses the estimation of both the uACR from urinalysis and eGFR from a blood test. Neither test alone can identify all individuals with T2D and CKD, which is why regularly undertaking both screening tests is important (15). However, in clinical practice, an annual eGFR is more commonly performed than the uACR (16, 17). If either screening test is positive (i.e. the uACR is significantly elevated (>30 mg/g) and/or the eGFR is significantly reduced (<60 mL/min/1.73 m^2^)), a diagnosis of CKD should be confirmed by repeat testing (15). It is seldom necessary to perform additional tests to define the cause of CKD in patients with T2D such as a kidney biopsy or other biomarkers. However, patients with an atypical presentation, such as a rapid onset of impaired kidney function, heavy proteinuria, microscopic haematuria or red blood cell casts in the urine or kidney pain, should be considered for referral to nephrology specialist services.

### Standard management approach for CKD

**Figure 1:**
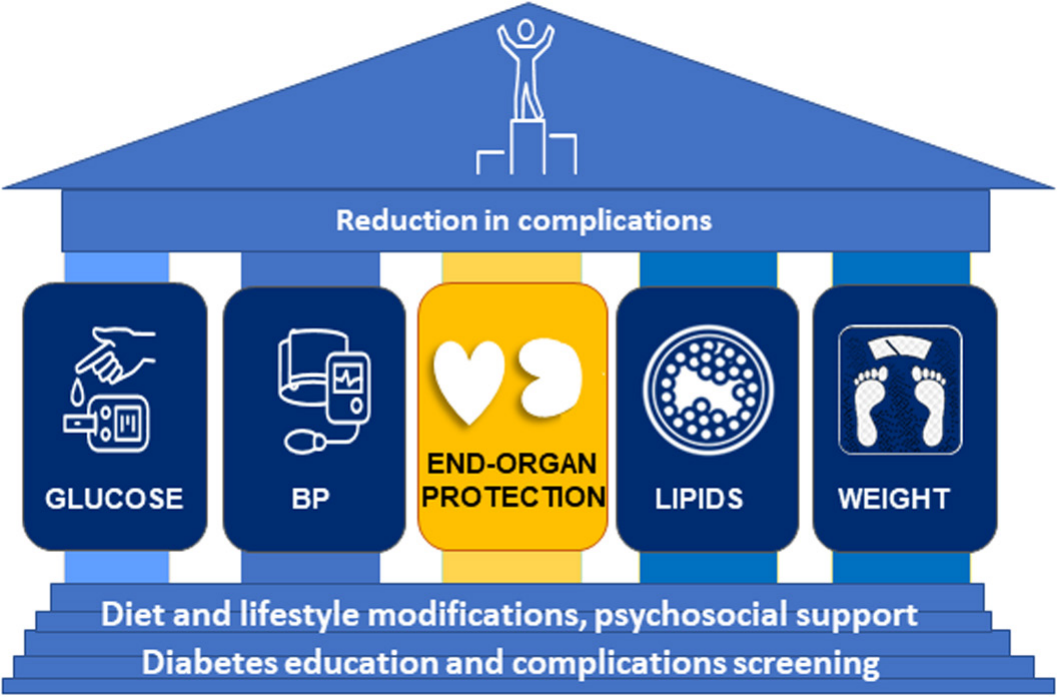
The multifactorial management of patients with T2D and CKD in which organ protection is now the central pillar of care, surrounded by residual risk factor reduction.

The true value of screening for CKD comes from the earlier opportunity it provides to initiate interventions to slow the decline in kidney function and reduce the incidence of impaired kidney function. As a rule of thumb, any treatment that slows the decline in eGFR by more than 0.75 mL/min/1.73 m^2^/year over 3 years predicts that the same intervention will also benefit hard kidney outcomes like the doubling of serum creatinine or end-stage kidney disease (ESKD) (18). Intensive multifactorial risk factor interventions remain the cornerstone of the prevention and management of CKD in people with T2D (15). This strategy includes optimal control of blood glucose, lipids, blood pressure and weight, as well as regular physical activity, a healthy diet and smoking cessation, built around a central pillar of organ protection (Fig. 1) (15). When instituted comprehensively in patients with T2D and CKD, this strategy can improve CV outcomes and survival (19). In addition, in STENO-2, kidney function decline was slower in the intensive-therapy group compared with the conventional-therapy group (3.1 mL/min/year vs 4.0 mL/min/year). Progression to ESKD was also numerically lower in the intensive group (hazard ratio (HR) 0.36; 95% confidence interval (CI) 0.12–1.05), although the study was underpowered to test this outcome (19).

Blockers of the renin–angiotensin–aldosterone system (RAAS), such as angiotensin-converting enzyme inhibitors, angiotensin receptor antagonists and mineralocorticoid receptor antagonists also have additional benefits in the kidney beyond simply lowering blood-pressure levels, particularly in patients with severely elevated albuminuria or impaired kidney function (20, 21, 22). However, in patients with T2D without CKD, any blood-pressure-independent renoprotective actions appear modest and limited to the prevention of new-onset macroalbuminuria (23). The use of high-potency statins in patients with CKD is also standard of care (15) because the CV risk of most people with T2D and CKD usually exceeds 10% over 10 years and is often considerably higher. However, although clearly beneficial for reducing major acute CV events (MACEs), statins do not prevent CKD and their effects on kidney function decline are not clinically significant in patients with CKD (24).

### Prognosis of CKD in patients with T2D

People with CKD are at increased risk of kidney failure, CVD, HF, infections, hospitalization and premature mortality (25). The most common causes of premature mortality in patients with T2D and CKD are atherosclerotic CVD, HF and infections. Sadly, most adults with T2D and CKD die prematurely due to these complications before progressing to kidney failure (26).

These clinical outcomes and prognosis in people with T2D are correlated to both the presence and the severity of CKD (27). For example, the larger the reduction in eGFR below 60 mL/min/1.73 m^2^, the greater the risk of poor health outcomes. Although the association between eGFR and outcomes is clearly continuous in nature (25), recent practice has been to more simply categorize patients by stages, like cancer. Stage 3a denotes an eGFR of 45–59 mL/min/1.73 m^2^, or moderately reduced kidney function; stage 3b denotes an eGFR of 30–44 mL/min/1.73 m^2^, or moderate to severely reduced kidney function; stage 4 denotes an eGFR of 15–29 mL/min/1.73 m^2^, or severely reduced kidney function and stage 5 denotes an eGFR of < 15 mL/min/1.73 m^2^, or kidney failure. Equally, the greater the urinary albumin excretion, the worse the prognosis (25). Again, recent practice has been to categorize patients based on the severity of urinary albumin excretion (A1: uACR < 30 mg/g, denoting normal to mildly increased albuminuria; A2: uACR 30–300 mg/g, denoting moderately increased albuminuria; and A3: uACR > 300 mg/g, denoting severely increased albuminuria) (15). These correspond to the previously used categories of normoalbuminuria, microalbuminuria and macroalbuminuria developed over 40 years ago. Individuals with both a reduced eGFR and an elevated uAER generally have a worse prognosis than individuals with either alone (27). This is conceptualized in the widely used prognostic chart adopted by the Kidney Disease: Improving Global Outcomes (KDIGO) guidelines for the management of CKD in people with T2D (15).

## SGLT2 inhibitors as the new standard of care for T2D and CKD

In addition to the standard of care interventions detailed earlier, it is now widely recommended that all people with T2D and CKD should be considered for treatment with an SGLT2 inhibitor (28). Indeed, recent KDIGO guidelines now consider SGLT2 inhibitors as first-line therapy, alongside metformin, for the management of patients with T2D and CKD (15, 29). This recommendation partly reflects the reduction in MACE outcomes and lower rates of hospitalization for HF consistently observed in the subgroup of patients with CKD randomized to receive an SGLT2 inhibitor in recent trials, including the CANagliflozin cardioVascular Assessment Study (CANVAS) Program (30), DECLARE-TIMI (31), DAPA-HF (32), EMPEROR-Reduced (33, 34) and EMPEROR-Preserved (35) (Supplementary Table 1, see section on supplementary materials given at the end of this article). Indeed, in these studies, the absolute beneficial effect of SGLT2 inhibitors on MACE outcomes and hospitalization for HF appears to be greatest in people with T2D and CKD (28, 36, 37).

Treatment with SGLT2 inhibitors in patients with T2D and CKD is also associated with a reduced incidence of CV death (HR 0.84; 95% CI 0.74–0.96) (36, 38), the single major cause of death in this setting. As a result, the risk of all-cause mortality was also reduced by 14% with SGLT2 inhibition treatment compared with placebo (HR 0.86; 95% CI 0.77–0.96) (36). In addition, these large trials have also documented (as an observational outcome) a clear slowing in the rate of decline in kidney function and reduced incidence of impaired kidney function (39), which has directly led to dedicated studies of SGLT2 inhibitors in people with CKD (detailed below).

### Recent trials undertaken in patients with CKD

Four large randomized controlled trials, each conducted against a backdrop of the standard of care, have been recently undertaken in participants with established CKD, some or all of whom also had T2D (Supplementary Table 1).

The CREDENCE study was the first study undertaken in individuals with T2D and established CKD, who were randomized to receive the SGLT2 inhibitor, canagliflozin or placebo, on top of a comprehensive standard of care (11). Most participants had a reduced eGFR and, of those who did not, all had severely elevated albuminuria, meaning that all participants were at very high risk of developing kidney failure. The CREDENCE trial was stopped early when it became clear at a prespecified interim analysis that participants receiving an SGLT2 inhibitor had benefits for the primary outcome (*P* < 0.01). At adjudication, the relative risk of the composite primary outcome (doubling of serum creatinine, dialysis, kidney transplantation or death from kidney or CV causes) was 30% lower in the participants receiving canagliflozin (HR 0.70; 95% CI 0.59–0.82; *P* = 0.00001). The key secondary kidney outcome (doubling of serum creatinine, end-stage kidney failure treated by dialysis or kidney transplantation, or death from kidney failure) was also reduced by 34% (HR 0.66; 95% CI 0.53–0.81; P < 0.001), and the relative risk of ESKD was lowered by 32% (HR 0.68; 95% CI 0.54–0.86; *P* = 0.002) with canagliflozin treatment compared with placebo (11).

The observed renoprotective effects of canagliflozin were consistent across all subgroups of the trial, including individuals with severely reduced eGFR (40). In addition, a *post hoc* analysis showed that canagliflozin treatment led to early sustained reductions in urinary albumin excretion, which were independently associated with improved long-term kidney and CV-related outcomes (41).

Finally, in the CREDENCE study, the rate of chronic decline of eGFR was also slowed by 60% following treatment with canagliflozin (42, 43). If this finding can be extrapolated into the future, the likelihood of developing kidney failure would have been delayed by an average of 13 years in participants receiving canagliflozin (42, 43).

The Dapagliflozin and Prevention of Adverse outcomes in Chronic Kidney Disease (DAPA-CKD) trial was undertaken in participants with established CKD, who were randomized to receive the SGLT2 inhibitor, dapagliflozin or placebo on top of standard of care. Approximately 68% of participants also had T2D (44, 45). Most participants had a reduced eGFR < 60 mL/min/1.73 m^2^ and elevated albuminuria (uACR > 200 mg/g), meaning that all participants were at high risk of developing kidney failure. The DAPA-CKD trial was also stopped early because of clear evidence of benefit at a prespecified interim analysis. The primary composite outcome (sustained decline in eGFR of ≥ 50%, kidney failure or death from kidney or CV causes) was reduced by 39% in participants receiving treatment compared with placebo (46). Specifically in participants with T2D, the primary outcome was reduced by 36% (HR 0.64; 95% CI 0.52–0.79) (13).

**Figure 2:**
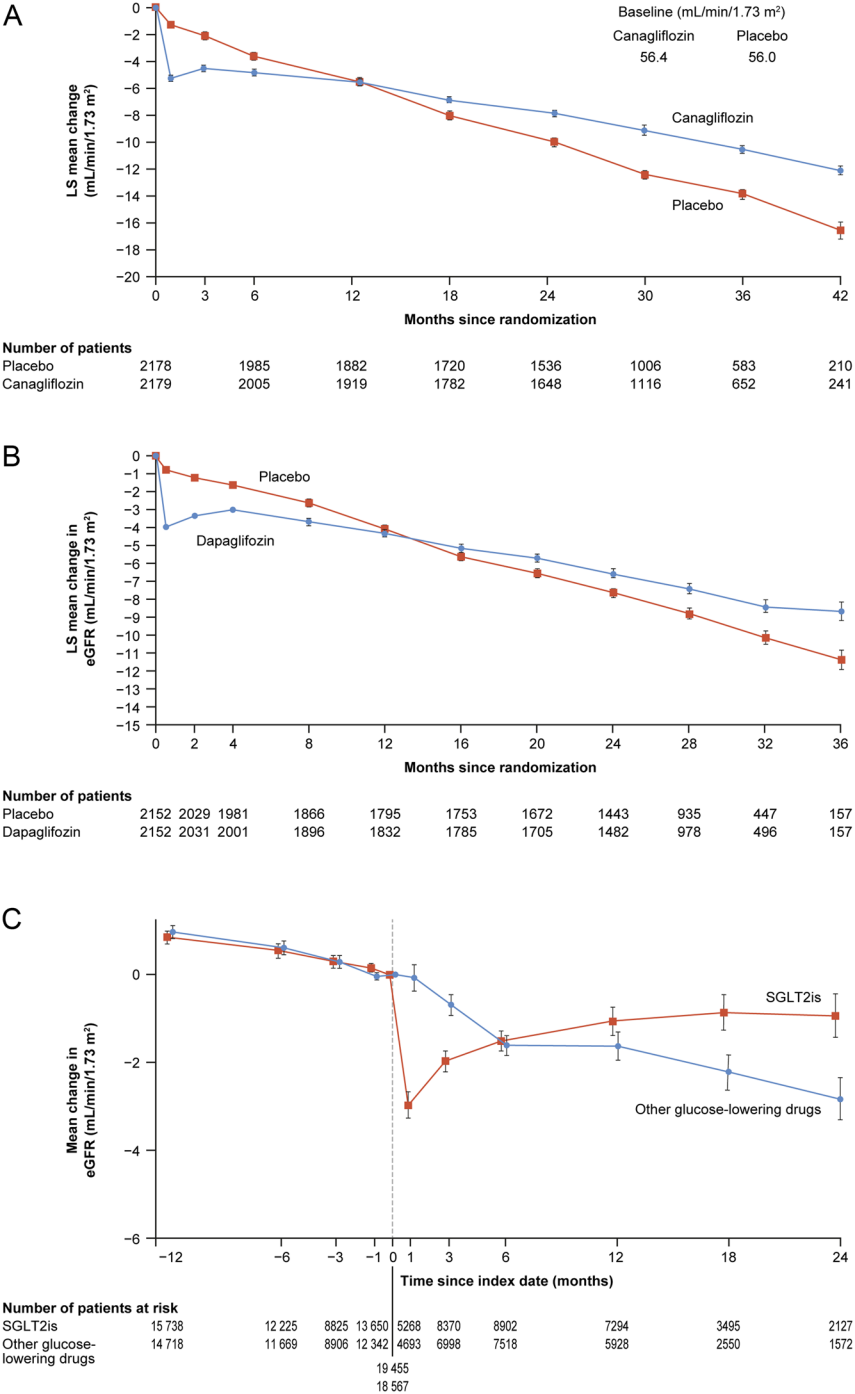
Changes from baseline in eGFR in patients treated with SGLT2 inhibitors vs placebo, against a background of standard of care, in two RCTs and one observational study. Panel A shows the LS mean change (s.e.) from baseline in the eGFR in patients treated with canagliflozin compared with placebo in the CREDENCE trial (data from 11). Panel B shows the LS mean change (s.e.) from baseline in the eGFR in patients treated with dapagliflozin compared with placebo in the DAPA-CKD trial (data from 46). Panel C shows the eGFR slope before and after initiation with an SGLT2 inhibitor or other glucose-lowering drug in the CVD-REAL-3 observational study (data from 47). eGFR, estimated glomerular filtration rate; LS, least-squares; RCT, randomized controlled trial; s.e., standard error; SGLT2, sodium-glucose co-transporter-2; SGLT2i, SGLT2 inhibitor.

The secondary kidney-specific composite outcome (sustained decline in eGFR of ≥50% or kidney failure, or death from kidney causes) was also reduced by 43% in participants with T2D treated with dapagliflozin (HR 0.57; 95% CI 0.45–0.73) and kidney failure was reduced by 30% (HR 0.69; 95% CI 0.51–0.92) (13). The rate of decline in eGFR was also slowed in participants with T2D (44) (dapagliflozin −1.58 mL/min/1.73 m^2^/year vs placebo: −3.84 mL/min/1.73 m^2^/year, equating to a 59% reduction in the rate of eGFR; Fig. 2) and fewer individuals with T2D developed a 50% decline in their eGFR during the study (13). In addition to actions on kidney function, treatment with dapagliflozin also showed a reduction in hospitalization for HF and all-cause mortality in participants with T2D and CKD (38).

In participants without T2D (i.e. with non-diabetic kidney disease) enrolled in the DAPA-CKD trial, the primary and secondary composite outcomes were also significantly improved and essentially no different from diabetic individuals (13). Notably, the major cause of non-diabetic CKD in this study was hypertension/renovascular disease, which is also more common in people with T2D and often also contributes to their kidney function decline in this setting. Taken together, these data show, for the first time, that SGLT2 inhibition is working to protect the kidneys, and not just working on the T2D that damages them.

The SCORED study assessed the safety of sotagliflozin, a dual SGLT1 and SGLT2 inhibitor, in 10,584 patients with T2D and impaired kidney function (eGFR 25–60 mL/min/1.73 m^2^) (14). Although undertaken in participants with CKD, its primary endpoint was changed during the trial to the composite of deaths from CV causes, hospitalizations for HF and urgent visits for HF. The trial was stopped early due to administrative reasons, including funding and the COVID-19 pandemic. Nonetheless, after a median follow-up of 16 months, the primary composite outcome was reduced by sotagliflozin compared with placebo (HR 0.74; 95% CI 0.63–0.88; *P* < 0.001). The MACE outcome (a composite of CV death, non-fatal myocardial infarction or stroke) was also reduced (HR 0.84; 95% CI 0.72–0.99), as was the composite outcome of death from CV causes or hospitalization for HF (HR 0.77; 95% CI 0.66–0.91) (14). The composite kidney endpoint (a sustained decrease in the eGFR of ≥50%, dialysis, kidney transplantation or a sustained eGFR of <15 mL/min/1.73 m^2^) was reduced by sotagliflozin but failed to reach significance (HR 0.71; 95% CI 0.46–1.08) with a low number of events limiting the power of the trial. The decline in eGFR was also stalled by sotagliflozin, while kidney function declined in the placebo group (sotagliflozin 0.09 mL/min/1.73 m^2^/year vs placebo −1.31 mL/min/1.73 m^2^/year) (14).

Finally, the EMPA-KIDNEY trial (12) assessed the effect of empagliflozin compared with placebo in over 6600 patients with CKD with or without diabetes (48). This study was also stopped after a prespecified interim review identified unequivocal efficacy. At adjudication, treatment with empagliflozin was associated with a statistically significant reduction in the primary composite outcome of kidney disease progression or CV death (HR 0.72; 95% CI 0.64 to 0.82; *P* < 0.001) (49). Reduction in all-cause hospitalization (HR 0.86; 95% CI 0.78, 0.95; *P* = 0.003) was also reported. Finally, the rate of decline in eGFR was significantly slowed following treatment with empagliflozin, with activity in all subgroups including participants without severely elevated albuminuria (49).

A recent meta-analysis incorporating all 13 placebo-controlled trials with SGLT2 inhibitors involving over involving 90,000 participants and using a standardized outcome definition for kidney disease progression (a sustained ≥50% eGFR decline from randomization, ESKD or death from kidney disease) concluded that treatment with an SGLT2 inhibitor reduced the risk of kidney disease progression by 37% (relative risk 0.63, 95% CI 0.58–0.69) (50). In the four CKD trials, positive effects were also reported on acute kidney injury, the risk of cardiovascular death and hospitalization for HF. Notably, these actions appear to be consistent irrespective of diabetes, kidney function or the primary cause of kidney disease. However, the absolute benefits of SGLT2 inhibitors appear greater (and the number needed to treat lower) in participants with CKD, and especially those with multimorbidity, due to higher risks.

### Observations from CVOTs with SGLT2 inhibitors

The kidney-protective effects of SGLT2 inhibitors described in trials in patients with CKD are further supported by consistent observations from other large trials with these agents (Supplementary Table 1). In particular, recent CVOTs undertaken in patients with T2D and established CVD/high CV risk (EMPA-REG OUTCOME, CANVAS Program, DECLARE-TIMI 58 and VERTIS-CV) included some individuals with established CKD (14, 51, 52, 53, 54). Although kidney outcomes were secondary or exploratory in these CVOTs, the subgroup of participants with T2D and CKD experienced similar relative improvements in kidney outcomes when treated with SGLT2 inhibitors to that documented in the CREDENCE study (55). Taken together, in the subgroup of participants with T2D and established CKD, there was a slowing in the rate of decline of kidney function and a reduced incidence of dialysis, transplantation or death due to kidney disease (relative risk (RR) 0.67; 95% CI 0.52–0.86, *P* = 0.0019) in participants receiving an SGLT2 inhibitor (55, 56). In addition, the effects of SGLT2 inhibition on CV and kidney outcomes observed in the EMPA-REG OUTCOME and CANVAS Program studies were consistent across the KDIGO risk categories (56, 57, 58). Although *post hoc*, these findings strongly support the favourable outcomes observed in patients with T2D and CKD. In addition, these CVOTs also observed a slower decline in eGFR and lower incidence of significant kidney function decline in participants without CKD (or ‘low risk’ using the KDIGO categorization system) (56, 57, 58). Although the absolute risk of kidney failure is low in these patients, its impact is severe and costly, meaning that primary prevention may still be beneficial for their kidneys. Of course, this is largely moot in patients with T2D and CVD, such as those participating in these trials, where their very high CV risk already mandates an aggressive approach to risk reduction, including consideration of SGLT2 inhibitors, potentially as first-line agents.

### Kidney benefits in randomized clinical trials in patients with HF

Evidence for the kidney-protective effects of SGLT2 inhibitors is further supported by consistent benefits observed in trials undertaken in patients with established HF. Of course, many of these trials included individuals with CKD because HF is a risk for CKD and vice versa. In addition, in this subgroup with CKD, the greatest absolute benefits were observed. For example, the DAPA-HF trial tested dapagliflozin, on top of the standard of care, in participants with chronic HF with reduced ejection fraction (HFrEF) (32). In this study, 41.8% had T2D, and 40% had an eGFR < 60 (9). DAPA-HF met its primary outcomes with a reduction in CVD death and hospitalization for HF. A key secondary outcome (the renal composite of a ≥50% sustained decline in eGFR, kidney failure or renal death) was modestly but not significantly reduced (0.71; 95% CI 0.44–1.16) (9). Although only nominally significant, as endpoints were exploratory, fewer patients experienced an eGFR decline of 30% (HR 0.68; 95% CI 0.58–0.79, *P* < 0.002), 40% (HR 0.54; 95% CI 0.43–0.67, *P* < 0.002) or 50% (HR 0.57; 95% CI 0.40–0.81, *P* < 0.002) to an eGFR < 60 mL/min/1.73 m^2^ with dapagliflozin compared with placebo. In addition, the rate of decline of eGFR (between day 14 and day 720) was significantly reduced with dapagliflozin treatment compared with placebo, as was the risk of the doubling of serum creatinine, a marker of kidney decline. Importantly, the effect of dapagliflozin on eGFR decline was independent of T2D status.

The EMPEROR-Reduced trial assessed the safety and efficacy of empagliflozin in patients with HFrEF, including half with diabetes and half with established CKD (33). In addition to meeting its primary outcome, the risk of the composite renal outcome (time to first event of chronic dialysis, renal transplantation or sustained reduction of eGFR of sustained ≥40%) was lower with empagliflozin treatment compared with placebo in patients with HFrEF (HR 0.50; 95% CI 0.32–0.77, *P* < 0.01) in the EMPEROR-Reduced trial (33, 34). This renal benefit effect was independent of diabetes status and baseline kidney function (33, 34). The rate of eGFR decline was also significantly slower with empagliflozin treatment compared with placebo (33).

The EMPEROR-Preserved trial assessed the safety and efficacy of empagliflozin in patients with HF with preserved ejection fraction (HFpEF), again including half with diabetes and half with established CKD (35, 59). In patients with HFpEF, there was no significant difference in the kidney-specific composite outcome (profound and sustained decreases in eGFR sustained ≥40% kidney-replacement therapy or kidney death; HR 0.95; 95% CI 0.73–1.24) in the EMPEROR-Preserved trial (59). But, again, empagliflozin slowed the decline in kidney function (35, 59). The reason for the different findings in participants with reduced or preserved kidney function in their respective trials is still unclear but may reflect the different kidney outcome definitions used. For example, when a consistent kidney outcome is used across all HF trials (sustained decreases in eGFR ≥ 50% kidney-replacement therapy or kidney death) the outcome of EMPEROR-Preserved (HR 0.78; 95% CI 0.54–1.13) is similar to that observed in DAPA-HF (HR 0.71; 95% CI 0.44–1.16) (60).

## Real-world studies with SGLT2 inhibitors

Results from real-world observational studies and registries indicate that the benefits of SGLT2 inhibitor therapy on kidney function seen in clinical trials are translatable to routine clinical practice. For example, the CVD-REAL-3 study was a multinational observational cohort study in patients with T2D, which compared outcomes in patients initiating SGLT2 inhibitors with those receiving other glucose-lowering drugs (47). The study showed that SGLT2 inhibitor was associated with a significant reduction in eGFR decline (difference in slope for SGLT2 inhibitors vs other glucose-lowering drugs of 1.53 mL/min/1.73 m^2^, 95% CI 1.34–1.72, *P* < 0.0001) and that, during follow-up (mean 14.9 months), patients taking SGLT2 inhibitors had a significantly lower rate of the composite kidney outcome (50% eGFR decline or kidney failure). These results were consistent across countries and prespecified subgroups (47). The EMPRISE study – an ongoing observational study in patients with T2D from two commercial and Medicare databases in the USA (2014–2019) – compared empagliflozin therapy with dipeptidyl peptidase-4 (DPP-4) inhibitor and glucagon-like peptide-1 receptor agonist (GLP1 RA) therapy, with a CV composite primary outcome and a kidney failure secondary outcome (61). Interim results (reported as of June 2021) showed that the risk of kidney failure was significantly reduced with empagliflozin treatment compared with DPP-4-inhibitor treatment (62).

## Safety and tolerability of SGLT2 inhibitors in patients with T2D and CKD

Patients with CKD generally have an increased risk of adverse drug reactions (ADRs) when starting new medications (63). This often leads to treatment inertia, even when the benefits of treatment appear unambiguous. Fortunately, SGLT2 inhibitors are generally well tolerated in patients with T2D and CKD. Nonetheless, many practitioners continue to have safety concerns around initiating SGLT2 inhibitors in patients with CKD and maintaining therapy below certain levels of kidney function. These are addressed below.

### SGLT2 inhibitor initiation and eGFR dip

Initiation of an SGLT2 inhibitor is often associated with a small ‘dip’ in eGFR of 3–6 mL/min/1.73 m^2^ (42, 64). This limited functional change does not appear to be harmful, and the CV and kidney outcomes of those experiencing a fall in eGFR appear to be no different to those experiencing a more modest change or no change in kidney function (65, 66). Moreover, tolerability and the risk of ADRs, including acute kidney injury (AKI), are not associated with the dip in eGFR following the initiation of an SGLT2 inhibitor (67). Finally, the dip also appears to be fully reversible upon discontinuation of SGLT2 inhibition.

Nonetheless, this dip in the eGFR is more common and marginally greater in patients with impaired kidney function who are already close to kidney failure. For example, the CREDENCE study showed that dips in eGFR of over 10% occurred in nearly half of all participants following the initiation of canagliflozin (65). Although this was not associated with adverse safety outcomes in a trial setting, in the real-world caution should still be exercised. While it is generally recommended that kidney function does not need to be monitored after initiation of an SGLT2 inhibitor in most patients with CKD, in individuals deemed at risk of AKI, review of hydration status and testing of kidney function 4 weeks after initiation has been suggested, although, in most cases, findings will not result in alteration of SGLT2 inhibition or dosing.

### Volume depletion

Initial glycosuria associated with SGLT2 inhibition can result in water loss and, at the same time, treatment with SGLT2 inhibitors reduces the plasma volume. In a small number of individuals, this may lead to symptoms of volume depletion (including hypotension, syncope and dehydration), although these symptoms are often mild and manageable with appropriate patient education and support (68). Diminished glycosuria in patients with CKD reduces polyuria. However, reductions in plasma volume and blood pressure are still observed in patients with impaired kidney function treated with SGLT2 inhibitors. Consequently, clinicians are advised to assess volume status prior to treatment initiation with SGLT2 inhibitors and to correct hypovolemia (particularly in elderly patients, in patients with impaired kidney function or low systolic blood pressure and in patients receiving diuretics) (69, 70, 71, 72). In most cases, symptoms are transient and subside as both plasma and urinary glucose levels fall. Starting SGLT2 inhibitors early (e.g. while haemoglobin A1c (HbA1c) < 8%) can also improve tolerability by limiting heavy glucosuria experienced by patients with poor control starting an SGLT2 inhibitor.

### Acute kidney injury

AKI is defined as an abrupt decrease in kidney function and is more common in people with CKD, especially those with diabetes (73). Following early case reports, drug labels for SGLT2 inhibitors include a warning about AKI and recommendations to minimize risk. However, data from recent randomized clinical trials (RCTs), meta-analyses and observational studies suggest that SGLT2 inhibitors do not support this risk, even in individuals with moderate-to-severely impaired kidney function. For example, in the DAPA-CKD trial, there was no significant difference in renal-related adverse events (AEs) or investigator-reported AKI-related serious AEs between treatment groups (46, 74), although abrupt declines in kidney function were fewer in participants receiving dapagliflozin (74). In the CREDENCE trial, there was no difference in AKI rates between the canagliflozin and placebo treatment groups (11). However, when taken together, data from recent CV and kidney outcome trials with SGLT2 inhibitors indicate that the risk of AKI is certainly not increased and may even be reduced by this therapy (RR 0.75; 95% CI 0.66–0.85, *P* < 0.0001) (57). Consistent with this observation, in a propensity-matched analysis using real-world data from two USA patient cohorts, SGLT2 inhibitors use reduced the risk of AKI by between 50 and 60% (unadjusted HRs) (75), and in the EMPRISE observational study, treatment with empagliflozin was associated with a 46% lower risk of AKI requiring dialysis (76). Whether this is a direct effect to protect the kidney or the indirect benefits of SGLT2 inhibition also reducing AKI-precipitant events, such as HF/volume decompensation, excessive diuretic use or MACE, remains to be established.

### Genital mycotic infections

The risk of a genital mycotic infection is approximately 3–4 times higher in patients taking an SGLT2 inhibitor compared with placebo due to the effects of glucose-containing urine on the perineal flora (77). Mycotic infections are most observed in women and some uncircumcised men. Although genital infections can be distressing, they are easily treated with topical antifungal ointments and/or oral medications without requiring discontinuation of SGLT2 inhibition (78). In most cases, genital infections can also be prevented by genital hygiene measures (e.g. washing after voiding and frequent pad changes). Early initiation of SGLT2 inhibition in people maintaining good control of their glucose levels is also advantageous because it reduces glycosuria and the risk of infection. For the same reasons, patients with T2D and CKD generally have reduced glycosuria, meaning the frequency of mycotic infection is lower in this setting, but it remains higher with SGLT2 inhibition when compared with placebo. Some studies have reported a lower risk of genital mycotic infection when SGLT2 inhibitors are used in combination with DPP-4 inhibitors, as is often done in patients with T2D and CKD. Whether this is through lower glucosuria or other mechanisms remains to be established. Fournier’s gangrene (an acute necrotic infection of the scrotum, penis or perineum) is a rarely reported ADR that has been potentially linked to SGLT2 inhibition in a small number of case reports. Most of these cases were also associated with poor genital hygiene, poor glucose control or inadequately treated mycotic infections, also contributing to skin barrier breakdown, making any causal association with SGLT2 inhibition unclear. In a meta-analysis by Staplin *et al.*, severe complications were rarely reported, with too few cases of Fournier’s gangrene to estimate risk ratios (77). Only one case of Fournier’s gangrene occurred in the DAPA-CKD study, and this occurred in the placebo group (46).

### Urinary tract infections

Patients with diabetes, and especially women with diabetes, have an increased risk of urinary tract infections (UTIs). For example, in the EMPA-REG OUTCOME trials over 30% of women in the placebo group experienced a UTI. However, this was not increased by using an SGLT2 inhibitor. The meta-analysis from Staplin *et al.* showed that there was only a non-significant 7% increase in the risk of UTIs in patients taking SGLT2 inhibitors (77). Similarly, in a population-based cohort study of more than 200,000 patients, there was no difference in the risk of UTI events between patients initiating SGLT2 inhibitors compared to those initiating DPP-4 inhibitors or GLP1 RA treatment (79). Even in those with a history of recurrent UTIs, treatment with dapagliflozin was not associated with an increased risk of UTI.

### Euglycaemic ketoacidosis

Euglycaemic ketoacidosis is a rare, but potentially life-threatening, complication of SGLT2 inhibition (80). In people with diabetes, SGLT2 inhibitor-induced glycosuria modestly increases blood ketone levels by increasing the glucagon:insulin ratio. This is similar in magnitude to the increase naturally observed in pregnancy and is not dangerous. However, as in pregnancy, superimposed stresses, such as starvation, dehydration, sepsis, and catabolism, can trigger dysregulated ketone production, leading to systemic acidosis. This is different to ‘classical’ diabetic ketoacidosis observed in type 1 diabetes, where inadequate insulin treatment fails to suppress both ketogenesis and blood glucose levels (leading to hyperglycaemia and profound dehydration).

Euglycemic ketoacidosis has been a very rare occurrence in RCTs. For example, in the DAPA-CKD trial, there were no reported cases of euglycemic ketoacidosis. In the CREDENCE study, rates of ketoacidosis were very low, but numerically higher in patients receiving canagliflozin (2.2 vs 0.2 per 1000 patient-years) (11). Overall, in 73,752 patients across all RCTs included in the meta-analysis by Staplin *et al.*, there were 159 ketoacidosis events, with the risk of a ketoacidosis event found to be approximately doubled in patients treated with an SGLT2 inhibitor compared with placebo, albeit off a very low baseline risk (77). No episodes of ketoacidosis were observed in people without diabetes in large SGLT2 inhibitor CKD or HF trials and only one case the recent EMPA-Kidney trial (50).

This risk for ketoacidosis can be simply mitigated by not using SGLT2 inhibitors in individuals with type 1 diabetes and ensuring that insulin doses are not omitted in insulin-treated patients with T2D. In addition, patients receiving SGLT2 inhibitors should be educated to pause therapy if they are not eating and drinking for long periods (e.g. they have gastroenteritis or prior to surgery) or they are unwell (e.g. they have flu COVID-19 or sepsis) (80, 81, 82, 83). Similarly, patients on SGLT2 inhibitors should be discouraged from initiation of ketogenic diets such as intermittent fasting or low-carbohydrate alternatives without adequate supervision. Patients undergoing major surgery should withhold SGLT2 inhibitor treatment preoperatively, and then only recommence postoperatively or post procedure once normal eating and drinking have been re-established. Studies are ongoing in patients admitted with acute coronary syndrome or acute HF to assess both the safety and efficacy of SGLT2 inhibition in this challenging setting.

### Electrolyte disturbance

Many people with T2D and CKD experience electrolyte disturbances, including hyperkalaemia, hyperphosphatemia, hypocalcaemia and hyponatremia. Importantly, the incidence of these dysfunctional changes is not increased following treatment with SGLT2 inhibitors. In the CREDENCE study, a reduced incidence of hyperkalaemia has been reported (HR 0.78, 95% CI 0.64–0.95, *P* = 0.014) (84). Indeed, a reduction in the incidence of serious hyperkalaemia (potassium > 6.0 mmol/L) of approximately 15% with SGLT2 inhibitors has been confirmed in a large individual participant data meta-analysis of people with T2D at high CV risk and/or with CKD (85).

### Severe hypoglycaemia

SGLT2 inhibitors lower blood glucose levels by causing urinary wasting of glucose. This mechanism is dependent on blood glucose levels and kidney function such that, if blood glucose levels return to the normal range, glycosuria is minimal. For this reason, these agents do not cause hypoglycaemia as monotherapy. In fact, the meta-analysis by Staplin *et al.* showed that the use of SGLT2 inhibitors actually reduced the relative risk of severe hypoglycaemia by 13% compared with placebo (77). This is partly due to the avoidance of hypoglycaemia-causing medications (e.g. insulin, sulphonylureas) achieved through glucose lowering with an SGLT2 inhibitor in people with T2D. It is advisable for clinicians to monitor glucose levels when SGLT2 inhibitors are given in combination with insulin, sulphonylureas or glinides, and, when necessary, reduce their doses. In such cases, only a slight adjustment is usually required, and patients should be reminded never to discontinue insulin while taking an SGLT2 inhibitor without first consulting their care provider. However, as the glycaemic effect diminishes as kidney function declines, in most patients with CKD stage 3 (eGFR < 60 mL/min/1.73 m^2^), it is seldom necessary to alter background glucose-lowering therapies when adding an SGLT2 inhibitor. Non-diabetic individuals treated with an SGLT2 inhibitor do not experience hypoglycaemia for the same reasons, and similarly, hypoglycaemia is not an issue for people with benign familial glycosuria; a genetic condition due to a mutation in *SGLT2*.

### Amputations

Foot disease in people with diabetes increases the risk of ulceration and lower-limb amputation. This risk is highest in people with T2D and CKD because the same processes that damage the kidneys also damage the nerves and vasculature supplying the feet. In the CANVAS Program, it was reported that the risk of lower-limb amputation was increased with canagliflozin treatment compared with placebo, especially in the first few months of treatment (30). The subsequent CREDENCE trial using the same agent showed no effect of canagliflozin, despite the higher risk of patients with CKD, and a pooled analysis of patient-level data from the CANVAS Program and CREDENCE trials concluded that the CANVAS Program amputation finding was most likely a chance effect (86). However, individuals with established foot disease were excluded from the CREDENCE trial due to a protocol amendment during the trial making interpretation problematic. None of the other SGLT2 inhibitors has reported an increased risk for amputation or other foot problems in their CVOTs, HF or kidney RCTs, making it unlikely that there is any drug class risk.

### Bone fractures

Bone fractures are a common and serious condition in older adults. Treatment of adults PPAR gamma agonists (e.g. pioglitazone) is associated with a significantly increased risk of major bone fractures. In the CANVAS Program, a potential signal for fracture risk was observed early in the study (87). However, this was likely a chance finding, and other trials with canagliflozin (e.g. CREDENCE) as well as a comprehensive systematic analysis of studies with other SGLT2 inhibitors, suggest that SGLT2 inhibitors are not associated with an increased risk of fracture (50). This is further supported by real-world data (88). Although mineral and bone disorder in advanced CKD is associated with an increased risk of bone fracture, recent studies of treatment of patients with CKD using SGLT2 inhibitors have not been with increased fracture risk.

### SGLT2 inhibitor dosing in patients with CKD

When SGLT2 inhibitors were first developed, their use was contraindicated in patients with impaired kidney function. This was because of the attenuated glucosuria in this setting and limited or no effects on blood glucose levels. Obviously, avoidance of SGLT2 inhibition in this setting has changed with the trial data detailed above demonstrating clear benefits for both kidney and cardiac outcomes in people with CKD, regardless of diabetes status. Currently, most guidelines recommend SGLT2 initiation down to an eGFR of 30 mL/min/1.73 m^2^ (15, 26). The US Food and Drug Administration now advises that dapagliflozin can be initiated down to an eGFR of >25 mL/min/1.73 m^2^ (69). Recent European guidelines have gone even lower and recommend dapagliflozin can be safely initiated down to an eGFR of 20 mL/min/1.73 m^2^ (15, 26). Should the eGFR level fall below these thresholds during therapy, treatment can continue, but new patients should not initiate SGLT2 inhibition below these eGFR levels at this time (mostly due to a lack of safety data in this setting). Future changes to KDIGO guidelines are likely to incorporate recommendations for lower eGFR limits for SGLT2 inhibitor use in CKD, with or without diabetes.

## Future directions

SGLT2 inhibitors have rapidly become first-line agents for the management of T2D complicated by CVD, by or CKD. However, for most of these patients, it is too late to restore normal organ function. The next important question is whether the early initiation of SGLT2 inhibitors will be able to prevent or slow the loss of function in the first place. Certainly, *de novo* admission for HF is reduced in patients using SGLT2 inhibitors. Future research is likely to investigate whether SGLT2 inhibitors can be used to prevent the development of CKD in people with diabetes (89, 90, 91). Mechanistically, the drivers of nephron dropout are similar in people both with and without CKD, although amplified in the latter due to reduced nephron numbers increasing nephron stress/vulnerability. Observational data from CVOTs suggest that patients without CKD not only experience a slower rate of decline in kidney function and a reduction in new-onset elevation in albuminuria (37). Indeed, the early initiation of SGLT2 inhibitors may offer the greatest kidney benefits in the long term (37, 92). Such findings strengthen the push to consider SGLT2 inhibitors as first-line treatment along with metformin for the management of all people with T2D if only to protect their kidneys.

Yet, despite these clear data, SGLT2 inhibitors remain seriously underutilized in clinical practice. Less than 10% of those with T2D and CKD are receiving SGLT2 inhibitors and even fewer in those with established CVD (93, 94). At the same time, at least ten times that number are receiving RAAS blockade and statin therapy. In the future, multifactorial therapy in patients with T2D will likely consider SGLT2 inhibition as an equal or more important part of preventing complications in diabetes, thereby reducing the progression of cardiorenal disease.

## Supplementary Materials

Supplementary Table 1 SGLT2i trials reporting renal and/or cardiovascular outcomes in patients with CKD or renal outcomes in patients with CVD.

## Declaration of interest

MCT has received honoraria for educational meetings performed on behalf of Boehringer Ingelheim, Lilly, MSD, Novartis, AstraZeneca, Mylan, Sanofi, & Servier; has received support from Boehringer Ingelheim; and has been an advisory board member for MundiPharma, AstraZeneca and MSD. MCT is a steering committee member for ‘Across Type 2 Diabetes’ and ‘Across CKD’ sponsored by Boehringer Ingelheim & Eli Lilly and Company Alliance. BLN has received fees for advisory boards, scientific presentations, steering committee roles and travel support from AstraZeneca, Bayer, Boehringer Ingelheim and Janssen, with all honoraria paid to his institution. SMT has attended advisory boards and received speaking fees from AstraZeneca, Abbott Diabetes Care and Nevro. MEC has received honoraria for educational symposia conducted on behalf of Boehringer-Ingelheim, Lilly, AstraZeneca, Abbott, Servier, Novartis, Sanofi, Bayer and MSD; has received support to attend and participate in advisory boards for Boehringer Ingelheim, Lilly, Bayer, Intarcia, MundiPharma and AstraZeneca; and has received research funding from Boehringer Ingelheim and Novo Nordisk. SVB has received speaking fees from Bayer, Pfizer and Vifor Pharma; has attended advisory boards for AstraZeneca, Bayer and Vifor Pharma; and has received non-financial research support from Bayer. All honoraria received were paid to SVB’s institution.

## Funding

Editorial support was provided by Emily Manktelow, PhD, of Oxford PharmaGenesis, Melbourne, Australia, and this support was funded by AstraZeneca http://dx.doi.org/10.13039/100004325.
