# Supplementary material for: SGLT2 inhibitors for patients with type 2 diabetes and CKD: a narrative review
Source: Endocr Connect. 2023 Jul 31;12(8):e230005. doi: 10.1530/EC-23-0005 (PMC10448577; doi:10.1530/EC-23-0005)
Supplement: Supplementary Table 1 SGLT2i trials reporting renal and/or cardiovascular outcomes in patients with CKD or renal outcomes in patients with CVD. [file supplementary_table_1.pdf]

Supplementary Table 1 SGLT2i trials reporting renal and/or cardiovascular outcomes in patients with CKD or renal outcomes in patients with CVD.

**Study name**<br>**NCT number**<br><br>Named 1° reference<br>(cited references)	**Population**	**Intervention and median follow-up**	**Baseline characteristics: SGLT2i (PBO)**	**Outcome**	**Result: SGLT2i vs PBO**
CREDENCE<br>(NCT02065791)<br><br>(Perkovic et al., 2019)<br><br>(Bakris et al., 2020a, Jardine et al., 2020, Oshima et al., 2021, Oshima et al., 2020a, Oshima et al., 2020b)	T2D with albuminuric CKD<br><br>(N = 4401)	Canagliflozin 100 mg (N = 2202) or PBO (N = 2199) once daily orally<br><br>2.6 years	Age (mean): 62.9 (63.2) years<br>Women: 34.6% (33.3%)<br>BMI (mean): 31.4 (31.3) kg/m^2<br>SBP (mean): 140 (140) mmHg<br>T2D duration (mean): 15.5 (16.0) years<br>HbA1c (mean): 8.3% (8.3%)<br>eGFR (mean): 56.3 (56) mL/min/1.73 m^2<br>uACR (median): 923 (931)	ESRD[a], doubling serum creatinine, or death from renal/CV causes (primary)<br><br>ESRD[a], doubling serum creatinine, or death from renal causes (secondary)<br><br>Dialysis, kidney transplantation or renal death (secondary)<br><br>CV death, myocardial infarction or stroke (secondary)<br><br>HHF (secondary)	43.2 vs 61.2 events/1000 p-yr<br>HR: 0.70 (95% CI: 0.59, 0.82)<br>*p* = 0.00001<br><br>27.0 vs 40.4 events/1000 p-yr<br>HR: 0.66 (95% CI: 0.53, 0.81)<br>*p* < 0.001<br><br>13.6 vs 18.6 events/1000 p-yr<br>HR: 0.72 (95% CI: 0.54, 0.97)<br>*p* = NA<br><br>38.7 vs 48.7 events/1000 p-yr<br>HR: 0.80 (95% CI: 0.67, 0.95)<br>*p* = 0.01<br><br>15.7 vs 25.3 events/1000 p-yr<br>HR: 0.61 (95% CI: 0.47, 0.80)<br>*p* < 0.001
DAPA-CKD<br>(NCT03036150)<br><br>(Heerspink et al., 2020d)<br><br>(Heerspink et al., 2021a, Heerspink et al., 2021b, Heerspink et al., 2020c, Jongs et al., 2021, Wheeler et al., 2020, Wheeler et al., 2021a)	CKD and stable dose of an ACE inhibitor or ARB for at least 4 weeks before screening<br><br>(N = 4304)	Dapagliflozin 10 mg<br>(N = 2152) or PBO (N = 2152) once daily orally<br><br>2.4 years	Age (mean): 61.8 (61.9) years<br>Women: 32.9% (33.3%)<br>BMI (mean): 29.4 (29.6) kg/m^2<br>SBP (mean): 137 (137) mmHg<br>T2D: 67.6% (67.4%)<br>HbA1c (mean): 7.1% (7.0%)<br>eGFR (mean): 43.2 (43.0) mL/min/1.73 m^2<br>uACR (median): 965 (934)	Decline in eGFR of ≥ 50%, ESRD[b], death from renal/CV causes (primary)<br><br>Decline in eGFR of ≥ 50%<br><br>ESRD<br><br>Death from renal causes<br><br>Decline in eGFR of ≥ 50%,	4.6 vs 7.5 events/100 p-yr<br>HR: 0.61 (95% CI: 0.51, 0.72)<br>*p* < 0.001<br><br>2.6 vs 4.8 events/100 p-yr<br>HR: 0.53 (95% CI: 0.42, 0.67)<br>*p* = NA<br><br>2.5 vs 3.8 events/100 p-yr<br>HR: 0.64 (95% CI: 0.50, 0.82)<br>*p* = NA<br><br>0.0 vs 0.1 events/100 p-yr<br>HR: —<br>*p* = NA<br><br>3.3 vs 5.8 events/100 p-yr

				ESRD[b] or death from renal causes (secondary)<br><br>Death from CV or HHF (secondary)	HR: 0.56 (95% CI: 0.45, 0.68)<br>*p* < 0.001<br><br>2.2 vs 3.0 events/100 p-yr<br>HR: 0.71 (95% CI: 0.55, 0.92)<br>*p* = 0.009
SCORED (NCT03315143)<br><br>(Bhatt et al., 2021a)	T2D, CKD with additional CV risk factors<br><br>(N = 10 584)	Sotagliflozin 200 mg, with an increase to 400 mg if unacceptable side effects did not occur (N = 5292), or PBO (N = 5292) once daily orally<br><br>16.0 months	Age (median): 69 (69) years<br>Women: 44.3% (45.5%)<br>BMI (median): 31.9 (31.7) kg/m^2<br>SBP (median): 138 (139) mmHg<br>HbA1c (median): 8.3% (8.3%)<br>eGFR (median): 44.4 (44.7) mL/min/1.73 m^2<br>uACR (median): 74 (75)	Deaths from CV causes, HHF and urgent visits for HF (primary)<br><br>Hospitalizations for HF and urgent visits for HF (secondary)<br><br>First occurrence of sustained decrease of ≥ 50% in eGFR from baseline for ≥ 30 days, long-term dialysis, renal transplantation or sustained eGFR of < 15 mL/min/1.73 m^2 (secondary)<br><br>Deaths from CV causes, nonfatal myocardial infarctions and nonfatal strokes (secondary)	5.6 vs 7.5 events/100 p-yr<br>HR: 0.74 (95% CI: 0.63, 0.88)<br>*p* < 0.001<br><br>3.5 vs 5.1 events/100 p-yr<br>HR: 0.67 (95% CI: 0.55, 0.82)<br>*p* < 0.001<br><br>0.5 vs 0.7 events/100 p-yr<br>HR: 0.71 (95% CI: 0.46, 1.08)<br>*p* = NA<br><br>4.8 vs 6.3 events/100 p-yr<br>HR: 0.77 (95% CI: 0.65, 0.91)<br>*p* = NA
EMPA-KIDNEY (NCT03594110) (The EMPA-KIDNEY Collaborative Group 2022 and 2023)	CKD with and without T2D<br><br>(N= 6609)	Empagliflozin 10 mg (N = 2997) or PBO (N = 2991) once daily orally<br><br>2.0 years	Age (mean): 63.8 years<br>Women: 33%<br>Diabetes: 46%<br>CVD 27%<br>SBP (mean): 136 mmHg<br>BMI : 29.7 kg/m2<br>eGFR (mean): 37.5 mL/min/1.73 m^2	Composite of progression of kidney disease (defined as ESKD, a sustained decrease in eGFR to <10 ml/min/1.73 m2, a sustained decrease in eGFR of ≥40%, or death from renal causes) or death from CV causes	HR 0.72 (95% CI 0.64, 0.82). p<0.001
EMPA-REG OUTCOME (NCT01131676)<br><br>(Wanner et al., 2016)<br><br>(Kraus et al., 2021, Zinman et al., 2015a)	T2D with CVD<br><br>(N = 7020)	Empagliflozin 10 mg or 25 mg (N = 3304) or PBO (N = 3305) once daily orally<br><br>3.1 years	Age (mean): 63.1 (63.2) years<br>Women: 29% (28%)<br>BMI (mean): 31 (31) kg/m^2<br>SBP (mean): 135 (136) mmHg<br>HbA1c (mean): 8.1% (8.1%)<br>eGFR (mean): 74.2 (73.8) mL/min/1.73 m^2	New onset albuminuria[c] (secondary)<br><br>New onset macroalbuminuria[d] (secondary)<br><br>Composite microvascular	51.5% vs 51.2%<br>HR: 0.95 (95% CI: 0.87, 1.04)<br>*p* = 0.25<br><br>11.2% vs 16.2%<br>HR: 0.62 (95% CI: 0.54, 0.72)<br>*p* < 0.001<br><br>14.0% vs 20.5%

				outcome[e] (secondary)<br><br>Incident or worsening nephropathy (secondary)<br><br>Initiation of renal-replacement therapy(secondary)<br><br>Doubling of serum creatinine level accompanied by eGFR ≤ 45 mL/min/1.73 m^2 (secondary)	HR: 0.62 (95% CI: 0.54, 0.70)<br>$p < 0.001$<br><br>12.7% vs 18.8%<br>HR: 0.61 (95% CI: 0.53, 0.70)<br>$p < 0.001$<br><br>0.3% vs 0.6%<br>HR: 0.45 (95% CI: 0.21, 0.97)<br>$p = 0.04$<br><br>1.5% vs 2.6%<br>HR: 0.56 (95% CI: 0.39, 0.79)<br>$p < 0.001$
EMPEROR-Reduced (NCT03057977)<br><br>(Packer et al., 2020a)<br><br>(Zannad et al., 2020b)	HFrEF<br><br>(N = 3730)	Empagliflozin 10 mg (N = 1863) or PBO (N = 1867) once daily orally<br><br>16.0 months	Age (mean): 67.2 (66.5) years<br>Women: 23.5% (24.4%)<br>BMI (mean): 28.0 (27.8) kg/m^2<br>SBP (mean): 123 (121) mmHg<br>T2D (mean): 49.8% (49.8%)<br>eGFR (mean): 61.8 (62.2) mL/min/1.73 m^2<br>LVEF (mean): 27.7% (27.2%)	Mean slope of change in eGFR (secondary)<br><br>Chronic dialysis or renal transplantation or a sustained reduction ≥ 40% eGFR or a sustained eGFR < 15 mL/min/1.73 m^2 in patients with a baseline eGFR ≥ 30 mL/min/1.73 m^2 or a sustained eGFR < 10 mL/min/1.73 m^2 in those with a baseline eGFR < 30 mL/min/1.73 m^2	−0.55 vs −2.28 mL/min/1.73 m^2<br>HR: 1.73 (95% CI: 1.10, 2.37)<br>$p < 0.001$<br><br>1.6 vs 3.1 events/100 p-yr<br>HR: 0.50 (95% CI: 0.32, 0.77)<br>p = NA
EMPEROR-Preserved (NCT03057951)<br><br>(Anker et al., 2021b)<br><br>(Zannad, 2021)	CHF with HFrEF, elevated NT-proBNP, structural HD within 6 months or HHF within 12 months<br><br>(N = 5988)	Empagliflozin 10 mg (N = 2997) or PBO (N = 2991) once daily orally<br><br>26.2 months	Age: 72 ± 9<br>Women 45%<br>Obese 45%<br>Diabetes 49%<br>CKD 50%<br>Hypertension 90%	Change in mean eGFR slope/year<br><br>Chronic dialysis or renal transplantation or a sustained reduction ≥ 40% eGFR or a sustained eGFR < 15 mL/min/1.73 m^2 in patients with a baseline eGFR ≥ 30 mL/min/1.73 m^2 or a sustained eGFR < 10 mL/min/1.73 m^2 in those with a baseline eGFR < 30 mL/min/1.73 m^2	−1.25 vs 2.62 mL/min/1.73 m^2<br>$p < 0.001$<br><br>3.6% vs 3.7% mL/min/1.73 m^2<br>$p > 0.05$
DECLARE-TIMI 58	T2D with CVD	Dapagliflozin 10 mg (N =	Age (mean): 63.9 (64.0) years	Sustained decrease of ≥	10.8 vs 14.1 events/1000 p-yr

(NCT01730534)<br><br>(Wiviott et al., 2019b)<br><br>(Mosenzon et al., 2019, Raz et al., 2018)	or multiple risk factors for atherosclerotic CVD<br><br>(N = 17 160)	8582) or PBO (N = 8578) once daily orally<br><br>4.2 years	Women: 36.9% (37.9%)<br>BMI (mean): 32.1 (32.0) kg/m^2<br>SBP (mean): 135 (135) mmHg<br>T2D duration (median): 11.0 (10.0) years<br>HbA1c (mean): 8.3% (8.3%)<br>eGFR (mean): 85.4 (85.1) mL/min/1.73 m^2	40% in eGFR to < 60 mL/min/1.73 m^2, ESRD or death from renal or cardiovascular causes (secondary)<br><br>Sustained decrease of ≥ 40% in eGFR to < 60 mL/min/1.73 m^2, ESRD or death from renal causes (secondary)	HR: 0.76 (95% CI: 0.67, 0.87)<br>*p* = NA<br><br>3.7 vs 7.0 events/1000 p-yr<br>HR: 0.53 (95% CI: 0.43, 0.66)<br>*p* = NA
DAPA-HF (NCT03036124)<br><br>(McMurray et al., 2019a)<br><br>(Jhund et al., 2021)	HFrEF<br><br>(N = 4744)	Dapagliflozin 10 mg (N = 2373) or PBO (N = 2371) once daily orally<br><br>18.2 months	Age (mean): 66.2 (66.5) years<br>Women: 23.8% (23.0%)<br>BMI (mean): 28.2 (28.1) kg/m^2<br>SBP (mean): 122 (122) mmHg<br>T2D: 41.8% (41.8%)<br>eGFR (mean): 66.0 (65.5) mL/min/1.73 m^2<br>LVEF (mean): 31.2% (30.9%)	Worsening renal function[f] (secondary)<br><br>Change from baseline to 8 months in creatinine (exploratory)	0.8 vs 1.2 events/100 p-yr<br>HR: 0.71 (95% CI: 0.44, 1.16)<br>*p* = NA<br><br>0.07 vs 0.04 mg/dL<br>HR: 0.02 (95% CI: 0.01, 0.03)<br>*p* < 0.007
DELIVER (NCT03619213)<br><br>(Causland et al. 2022)	HF with EF>40%<br><br>(N = 6263)	Dapagliflozin 10mg (N=3131) or PBO (N=3132) once daily orally<br><br>2.3 years	Age: 72 ± 10 years;<br>44% women;<br>45% BMI > 3o kg/m2<br>45% type 2 diabetes mellitus;<br>57% with history of atrial fibrillation or flutter).<br>LVEF (mean) 54.2 ± 8.8% .	eGFR slope (prespecified)<br><br>post hoc composite kidney outcome (first ≥50% decline in eGFR from baseline; first eGFR <15 mL/min/1.73 m2; ESKD,or death from kidney causes	Slower eGFR decline from month 1 to 36 (difference, 1.4; 95% CI, 1.0-1.8) mL/min/1.73 m2 per year; P < .001)<br><br>Non effect on kidney composite outcome (HR, 1.08; 95% CI, 0.79-1.49).
CANVAS program (NCT01032629 and NCT01989754)<br><br>(Neal et al., 2017)<br><br>(Neuen et al., 2018, Perkovic et al., 2018)	T2D with high CVD risk<br><br>(N = 10 142)	Canagliflozin 100 or 300 mg (N = 4347) or PBO (N = 5795) once daily orally<br><br>126.0 weeks	Age (mean): 63.2 (63.4) years<br>Women: 35.1% (36.7%)<br>BMI (mean): 31.9 (32.0) kg/m^2<br>SBP (mean): 136 (137) mmHg<br>T2D duration (mean): 13.5 (13.7) years<br>HbA1c (mean): 8.2% (8.2%)<br>eGFR (mean): 76.7 (76.2) mL/min/1.73 m^2<br>uACR (median): 12.4 (12.1)<br>History of nephropathy: 17.2% (17.9%)	Progression of albuminuria[g] (secondary)<br><br>Reduction by 40% in eGFR, renal replacement therapy or renal death (exploratory)<br><br>Regression of albuminuria (exploratory)<br><br>Sustained doubling of serum creatinine, ESRD, death from renal causes	89.4 vs 128.7 events/1000 p-yr<br>HR: 0.73 (95% CI: 0.67, 0.79)<br>*p* = NA<br><br>5.5 vs 9.0 events/1000 p-yr<br>HR: 0.60 (95% CI: 0.47, 0.77)<br>*p* = NA<br><br>293.4 vs 187.5 events/1000 p-yr<br>HR: 1.70 (95% CI: 1.51, 1.91)<br>*p* = NA<br><br>1.5 vs 2.8 events/1000 p-yr<br>HR: 0.53 (95% CI: 0.33, 0.84)<br>*p* = NA
VERTIS CV (NCT01986881)	T2D with established atherosclerotic	Ertugliflozin 5 mg or 15 mg (N = 5499) or PBO (N = 2747) once daily orally	Age (mean): 64.4 (64.4) years<br>Women: 29.7% (30.7%)<br>BMI (mean): 31.9 (32.0) kg/m^2	Death from renal causes, renal replacement dialysis/transplant, or	0.9 vs 1.2 events/100 p-yr<br>HR: 0.81 (95% CI: 0.63, 1.04)<br>*p* = NA

(Cannon et al., 2020)<br><br>(Cannon et al., 2018, Cherney et al., 2021a, Cherney et al., 2021b)	CVD<br><br>(N = 8246)	3.5 years	SBP (mean): 133.5 (133.1) mmHg<br>T2D duration (mean): 12.9 (13.1) years<br>HbA1c (mean): 8.2% (8.2%)<br>eGFR (mean): 76.1 (75.7) mL/min/1.73 m^2	doubling of the serum creatinine level (secondary)<br><br>Sustained 40% decrease in eGFR, renal dialysis/transplant, or renal death (pre-specified exploratory)	6.0 vs 9.0 events/1000 p-yr<br>HR: 0.66 (95% CI: 0.50, 0.88)<br>*p* = NA
SOLOIST-WHF (NCT03521934)<br><br>(Bhatt et al., 2020)	T2D recently hospitalized for worsening HF<br><br>N = 1222	Sotagliflozin 200 mg (increase to 400 mg depending on side effects) (N = 608) or PBO (N = 614) once daily orally<br><br>9.0 months	Age (median): 69 (70) years<br>Women: 32.6% (34.9%)<br>BMI (median): 30.4 (31.1) kg/m^2<br>SBP (median): 122 (122) mmHg<br>HBA1c (median): 7.1% (7.2%)<br>eGFR (median): 49.2 (50.5) mL/min/1.73 m^2	Change in eGFR (secondary)	–0.34 vs –0.18 mL/min/1.73 m^2<br>Difference in LS mean change: –0.16 (95% CI: –1.30, 0.98)<br>*p* = NA

[a]Defined as eGFR < 15 mL/min/1.73 m^2, dialysis initiated and kidney transplantation.

[b]Defined as eGFR < 15 mL/min/1.73 m^2, long-term dialysis and kidney transplantation.

[c]Defined as uACR ≥ 30 mg/g.

[d]Defined as uACR ≥ 300 mg/g.

[e]Composite microvascular outcome defined as initiation of retinal photocoagulation, vitreous haemorrhage, diabetes-related blindness, or new or worsening nephropathy (defined as new onset of macroalbuminuria; or doubling of serum creatinine level accompanied by an eGFR [based on modification of diet in renal disease formula] ≤ 45 mL/min/1.73 m^2; or initiation of continuous renal replacement therapy; or death due to renal disease).

[f]Defined as a sustained decline in the eGFR ≥ 50%, ESRD (defined as a sustained [≥ 28 days] eGFR of < 15 mL/min/1.73 m^2, sustained dialysis or renal transplantation) or renal death.

[g]Defined as a > 30% increase in albuminuria and a change from either normoalbuminuria to microalbuminuria or macroalbuminuria, or from microalbuminuria to macroalbuminuria.

NA denotes not applicable because *p* values are reported only for outcomes that were included in the hierarchical-testing strategy.

ACE, angiotensin-converting enzyme; ARB, angiotensin receptor blocker; BMI, body mass index; CHF, chronic heart failure; CI, confidence interval; CKD, chronic kidney disease; CV, cardiovascular; CVD, cardiovascular disease; eGFR, estimated glomerular filtration rate; ESRD, end-stage renal disease; HbA1c, haemoglobin A1c; HD, heart disease; HF, heart failure; HFrEF, heart failure with reduced ejection fraction; HHF, hospitalization for heart failure; HR, hazard ratio; LS, least-squares; LVEF, left ventricular ejection fraction; NT-proBNP, N-terminal pro B-type natriuretic peptide; PBO, placebo; p-yr, person-years; SBP, systolic blood pressure; SGLT2i, sodium–glucose cotransporter 2 inhibitor; T2D, type 2 diabetes; uACR, urine albumin–creatinine ratio.
